# Estimating the Burden and Distribution of Post–COVID-19 Condition in Washington State, March 2020–October 2023

**DOI:** 10.5888/pcd21.230433

**Published:** 2024-06-27

**Authors:** Arran Hamlet, Daniel Hoffman, Sharon Saydah, Ian Painter

**Affiliations:** 1 Epidemic Intelligence Service, Division of Workforce Development, Centers for Disease Control and Prevention, Atlanta, Georgia; 2 Washington State Department of Health, Shoreline, Washington; 3 Coronavirus and Other Respiratory Viruses Division, National Center for Immunization and Respiratory Diseases, Centers for Disease Control and Prevention, Atlanta, Georgia

## Abstract

**Introduction:**

After SARS-CoV-2 infection, some people will experience long-term sequelae known as post–COVID-19 condition (PCC). Although PCC is recognized as a public health problem, estimates of the prevalence of PCC are sparse. We described a framework for estimating the incidence and prevalence of PCC by population subgroups and geography over time in Washington State.

**Methods:**

We collected data on reported COVID-19 cases and hospitalizations and estimated SARS-CoV-2 infections in Washington State from March 2020 through October 2023. The reported case data were incorporated with parameter estimates from published articles and prevalence estimates from the Household Pulse Survey into a mathematical compartmental model of PCC progression. The model used differential equations to describe how the population of people with PCC moved through the model’s various stages. This framework allowed us to integrate data on age group, sex, race and ethnicity, vaccination status, and county to estimate incidence and prevalence of PCC for each subgroup.

**Results:**

Our model indicated that 6.4% (95% CI, 5.9%–6.8%) of all adults in Washington State were experiencing PCC as of October 2023. In addition to temporal differences in PCC prevalence and incidence, we found substantial differences across age groups, race and ethnicity, and sex. Geographic heterogeneity was pronounced, with the highest rates of PCC in central and eastern Washington.

**Conclusion:**

Estimation of PCC prevalence is essential for addressing PCC as a public health problem. Responding to PCC will require continued surveillance, research, and dedicated financial and public health action. This analysis, accounting for heterogeneities, highlights disparities in the prevalence, incidence, and distribution of PCC in Washington State and can better guide awareness and response efforts.

**Summary**

**What is already known about the topic?**

After SARS-CoV-2 infection, some people will have post–COVID-19 condition (PCC). Although PCC is widely recognized as a substantial public health problem, estimates of the prevalence of PCC are sparse.

**What is added by this report?**

An estimated 6.4% (95% CI, 5.9%–6.8%) of adults in Washington State had PCC as of October 2023. The prevalence of PCC varied substantially by county, age group, sex, and race and ethnicity.

**What are the implications for public health practice?**

Although the population prevalence of PCC has varied over time, it remains elevated. Geographic and subgroup differences in prevalence highlight the need for tailored approaches rather than a blanket statewide policy.

## Introduction

Between its emergence in December 2019 and October 2023, SARS-CoV-2 resulted in approximately 772 million reported cases and 6.98 million reported deaths worldwide (1). The total number of infections and deaths is likely substantially higher (2). Despite nonpharmaceutical interventions, effective vaccines and therapeutics, and naturally acquired immunity, COVID-19 remains a leading cause of death in the US (3).

For people who survive SARS-CoV-2 infection, some will go on to experience long-term sequelae, or post–COVID-19 condition (PCC). Rather than a single condition, PCC represents a collection of conditions and syndromes that affect almost every bodily system and includes more than 50 different symptoms (4,5). These new, returning, or ongoing health problems in the months after the acute stage of COVID-19 might last for weeks, months, or years (6,7). Although some symptoms are shared with other postviral syndromes, multiple symptoms appear to be specific to SARS-CoV-2 infection (8). The effects of PCC can range from mild to severe (9).

Although the prevalence of PCC is higher among people with severe disease and first infections, even mild cases and reinfections can lead to PCC (10,11). Because relatively minor symptoms often go unreported and access to testing for SARS-CoV-2 is not universal, a portion of people with PCC might not have a confirmed and reported SARS-CoV-2 infection (12). Estimating the prevalence of PCC is challenging because of the changing characteristics of acute SARS-CoV-2 variants (13), the effects of vaccinations and acute COVID-19 treatments (14,15), the occurrence of reinfections, and the range of characteristics of PCC (16).

Multiple methods have been used to overcome the difficulties associated with estimating the population-level prevalence and incidence of PCC, including surveys (6,7), machine learning techniques (17), and statistical and mathematical modeling (18,19). Although statistical and mathematical analyses might help overcome some limitations, the foundation for these sophisticated approaches is accurate, timely, and complete data. To this end, the Centers for Disease Control and Prevention (CDC) supports surveillance efforts to gather point estimates of PCC prevalence and information on how PCC affects US communities (7,16). These efforts to generate primary data are invaluable for estimating the effect of PCC on populations (5).

However, these estimates are available only at the state level. Demography, the proportion of the population living in rural areas, vaccination rates, and aspects of COVID-19 transmission (eg, rates, mode) vary substantially on a county-by-county basis. It may be misleading, and unhelpful, to try to apply a state average to individual counties. Furthermore, in Washington State, health decisions are made at the local health jurisdiction level, not the state. To carry out actionable and useful public health interventions, an understanding of the burden of PCC at the county level is warranted.

We developed a framework for estimating the PCC burden and applied the framework to data from Washington State. We used existing COVID-19 surveillance systems and estimates from the literature to set parameters for a descriptive compartmental model of PCC progression. This approach allowed us to extrapolate beyond the coarse estimates provided by external surveys and produce monthly county-specific estimates, categorized by age, sex, and race and ethnicity. By building on preexisting studies and data sources we can estimate both the incidence and burden of PCC across the state at a county level. This granular information is vital for advocacy and coordinating public health action and response in Washington State.

## Methods

### Descriptive compartmental model

**Figure 1:**
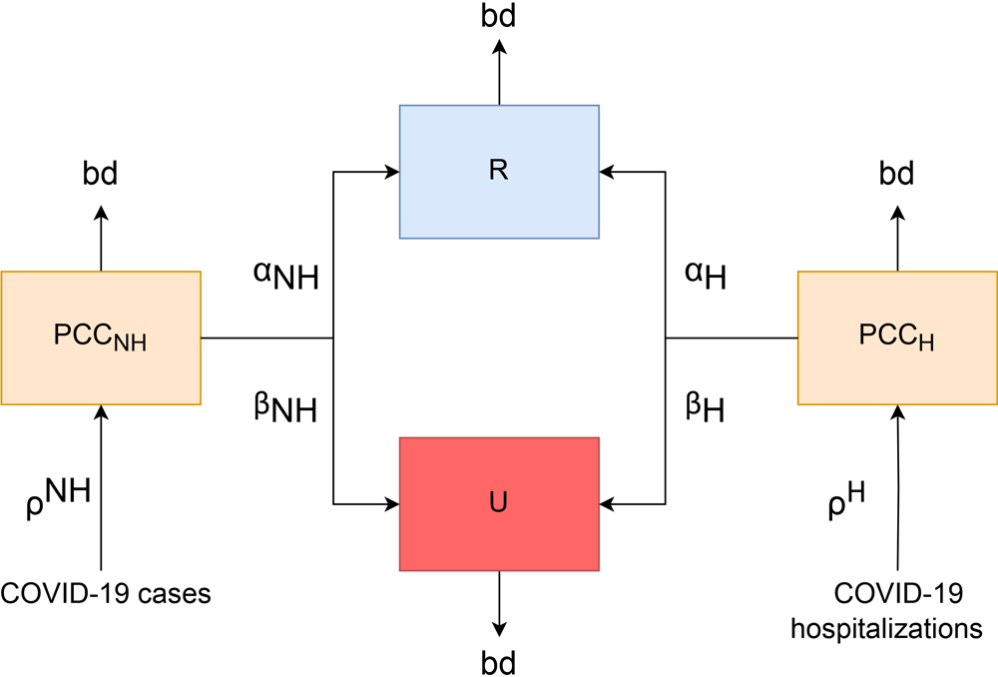
Diagram of the mathematical model for the progression of post–COVID-19 condition (PCC). Abbreviations: bd, background death rate; H, hospitalized; NH, nonhospitalized; R, recovered; U, unrecovered; α, the proportion moving to the recovered compartment; β, the proportion moving to the unrecovered compartment; ρ, the probability of a case or hospitalization developing PCC.

We used a compartmental model to estimate the burden of PCC (Figure 1). For this study, we defined burden as the incidence and prevalence of PCC, which we specified first as a statewide value and then by county, age group, sex, and race and ethnicity. The use of a compartmental model and data from the Household Pulse Survey (7) allowed us to take estimates for Washington State as a whole and extrapolate to the county level. This project was reviewed by CDC and determined to be nonresearch because it was an evaluation of a public health problem.

This model shows how people with COVID-19 may progress to PCC and then either recover or continue to exhibit symptoms. Hospitalized (denoted by _H_) and nonhospitalized (_NH_) people with PCC follow different probabilities and rates. Estimates of the reported number of PCC cases among nonhospitalized people are entered into the PCC_NH_ compartment and estimates of PCC among hospitalized patients are entered into the PCC_H_ compartments. These people are assigned to these compartments for 3 months, per our definition. They either progress to recovered (R) at probability α_H_ and α_NH_ or to the unrecovered (U) compartment at probability β_H_ and β_NH_, respectively. All compartments have a background death rate (bd). This is the monthly probability of death, unique for each age group (20). Each compartment contains multiple subcompartments (age group, sex, and race and ethnicity). Age has 9 subcompartments (0–17, 18–29, 30–39, 40–49, 50–59, 60–69, 70–79, ≥80, and unknown); sex has 3 subcompartments (male, female, unknown) and race and ethnicity have 6 subcompartments (Hispanic of any race, non-Hispanic Asian, non-Hispanic Black, non-Hispanic White, non-Hispanic ≥2 races or other race, and unknown). Race and ethnicity categories were specified in the Household Pulse Survey (7).

### Model equations

We fit a series of differential equations to model the transitions between the different compartments and subcompartments. This equation represents PCC cases among nonhospitalized people and their progression to either the recovered or unrecovered compartment:

$\frac{d{PCC}_{ijklm}^{NH}}{dt}={\rho_{ijklm}^{NH}COVID19\;Cases}_{ijklm}-\;{PCC}_{ijkl}^{NH}\left({bd}_{ij})-{PCC}_{ijkl,t\;-\;3}^{NH}(\;{\gamma^{NH}\alpha }_{ijkl}^{NH}+\;\beta_{ijkl}^{NH}\right)$

Where *i* is the 9 age groups, *j* the 3 sex categories, *k* the 6 race and ethnicity groups, *l* the 4 vaccination categories (unvaccinated, single dose, ≥2 doses, and unknown), and *m* the 40 counties (including unknown). PCC cases leaving the compartment are delayed for 3 months, per our definition of PCC. And where ρ^NH^ is the probability of a person developing PCC and COVID19 Cases is the number of cases reported to the Washington State Department of Health and the estimated symptomatic SARS-CoV-2 infections. The background death rate, *bd_ij_*, the recovery rate, γ^NH^, the probability of recovering, $\alpha_{ijkl}^{NH}$ to the recovered compartment (R) and the probability of moving to the unrecovered compartment U, $\beta_{ijkl}^{NH}$ control movement out of the compartment. The value of the PCC compartment from 3 months ago, per definition of PCC, is used.

PCC cases among hospitalized people progress to either recovered or unrecovered.

$\frac{d{PCC}_{ijklm}^{H}}{dt}={\rho_{ijklm}^{H}COVID19\;Hospitalizations\;}_{ijklm}-\;{PCC}_{ijklm}^{H}\left({bd}_{ij})-\;{PCC}_{ijklm,t-3}^{H}(\;{\gamma^{H}\alpha }_{ijkl}^{H}+\;\beta_{ijkl}^{NH}\right)$

ρ^H^ is the probability of a person developing COVID-19, and COVID19 Hospitalizations the number of COVID-19 hospitalizations reported to the Washington State Department of Health. The transition into the U and R compartments uses previously described parameters as follows:

$\frac{{dU}_{ijklm}}{dt}=\;{{PCC}_{ijklm}^{NH}\beta }_{ijkl}^{NH}+\;{{PCC}_{ijklm}^{H}\beta }_{ijkl}^{H}-\;U_{ijklm}{bd}_{ij}$

$\frac{{dR}_{ijklm}}{dt}=\;{PCC}_{ijklm}^{NH}\alpha_{ijkl}^{NH}+\;{PCC}_{ijklm}^{H}\alpha_{ijkl}^{H}-\;R_{ijklm}{bd}_{ij}$

For both these compartments, at different rates, PCC cases enter from the hospitalized and nonhospitalized compartment and leave through the background death rate.

### Definition of PCC

PCC has multiple definitions (21). We used the definition from the Household Pulse Survey, which is having any symptom lasting 3 months or more that was not present before having coronavirus or COVID-19 (7). This definition does not rely on a laboratory diagnosis of COVID-19.

### Data from the Household Pulse Survey

The US Census Bureau, with input from multiple federal agencies, collects information on PCC symptoms and effects through the Household Pulse Survey (7). This survey assesses the social and economic effects of emergent issues on US households. The sampling pool includes 130,220,000 households, which are sampled to produce demographically representative estimates. To ensure statistical rigor, several weightings and adjustments are applied.

The survey provides estimates of the proportion of the population experiencing PCC symptoms by age group, sex, race and ethnicity, and state. We used the survey’s output of people as “The percentage of adults who EVER experienced post-COVID conditions (long COVID) among those who ever had COVID” in Washington State, disaggregated by age group, sex, and race and ethnicity, as probabilities of having PCC. The Household Pulse Survey outputs also contain information on the percentage of people who have “significant activity limitations (‘yes, a lot’ response) from long COVID, among adults who are currently experiencing long COVID and among all adults.” We multiplied these percentages by the model estimates for PCC to estimate the percentage of the population that has significant activity limitations resulting from PCC.

### Reported cases, unreported cases, and hospitalization data

Individual-level data (eg, demographic information, vaccination status) on reported COVID-19 cases and people hospitalized with COVID-19 were obtained from the Washington Disease Reporting System, an electronic disease surveillance system. As a notifiable condition, COVID-19 cases are subject to mandatory reporting.

We calculated unreported cases of COVID-19 by using the time-varying estimates of total SARS-CoV-2 infections, provided by the mathematical model developed by the Institute for Disease Modeling and used by the Washington State Department of Health (22). This model takes into account data such as test positivity, case and hospitalization rates, and variant-specific effects to estimate overall SARS-CoV-2 infections, not just those captured by the surveillance system. We estimated SARS-CoV-2 infections at the state level. We subtracted the number of reported COVID-19 cases and hospitalizations from the number of estimated symptomatic SARS-CoV-2 infections to provide an estimate of uncounted COVID-19 cases. We multiplied the estimated number of uncounted COVID-19 cases by the percentage of people expected to be symptomatic (91.6% before the Omicron variant was dominant and 74.5% when it was dominant in Washington State [January 2022 onwards]) (23,24) to estimate the number of symptomatic infections not reported to the Washington Disease Reporting System. We assigned these uncounted COVID-19 cases to the “unknown” subcompartments of the case data.

**Figure 2:**
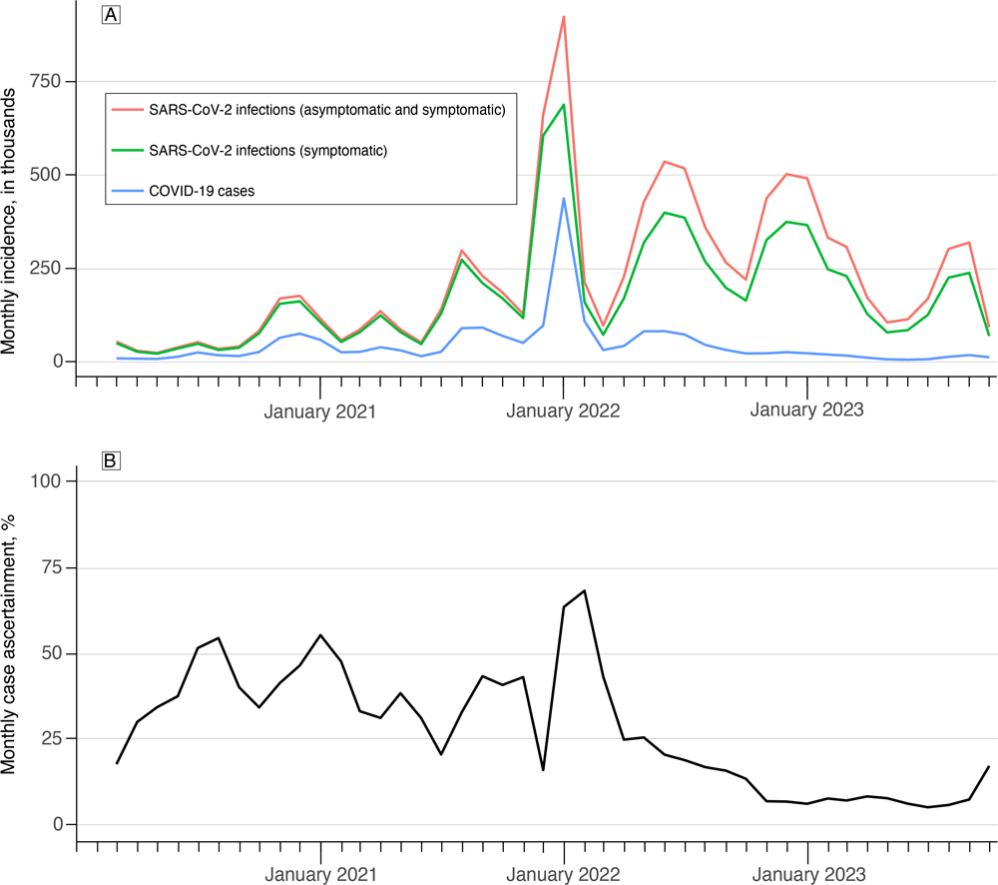
SARS-CoV-2 infections, COVID-19 cases, and COVID-19 case ascertainment in Washington State, March 2020–October 2023. A) Estimated number of SARS-CoV-2 infections (asymptomatic and symptomatic), estimated number of symptomatic SARS-CoV-2 infections, and reported number of COVID-19 cases. B) Case ascertainment calculated as the reported number of COVID-19 cases divided by the estimated number of symptomatic SARS-CoV-2 infections. Data source: Washington Disease Reporting System.

The Washington State Department of Health modeling group estimated that overall, only 20% of symptomatic infections are reported, with this proportion changing substantially monthly (Figure 2). Because of the large amount of missing information, we calculated age group, sex, and race and ethnicity breakdowns of model estimates as relative estimates at the final time point (defined as October 2023) rather than the absolute prevalence. We calculated the relative estimates of prevalence by dividing an individual subgroup (for age, one of the age categories) prevalence estimate by the median value of the group (ie, all age groups). To normalize prevalence estimates for the county-level map and heatmap, we use the following formula: $z_{i}=\;\frac{x_{i}-min(x)}{\max\left(x\right)-min(x)}$ where *i* represents the individual subgroup, *z* the normalized prevalence estimate, and *x* the group.

### Demographic data

We obtained data on population by race and ethnicity, age category, and county from the Washington Office of Financial Management (25) and data on life expectancy by age from the Office of the Insurance Commissioner of Washington State (20). In Washington State, as of 2015, on average a person living with a disability required $15,068 to cover annual health care costs (26).

To estimate the potential economic effect of PCC on our study population, we multiplied the aforementioned annual health care costs by the predicted incidence of PCC, which had been scaled according to the Household Pulse Survey’s estimates of the percentage of significant activity limitations resulting from PCC.

### Transition probabilities and rates

To approximate the prevalence estimates provided by the Household Pulse Survey, we used the least-squares method in the optimr R package (27) to fit 4 parameters: 1) the recovery rate for people with nonhospitalized PCC, 2) the multiplier applied to provide the recovery rate for people hospitalized with PCC, 3) the proportion of nonhospitalized PCC cases that move to the unrecovered compartment, and 4) the proportion of hospitalized PCC cases that move to the unrecovered compartment. We obtained data on the proportions of the population with PCC by age group, sex, and race and ethnicity from the Household Pulse Survey (7). Of 1,878,575 records of COVID-19 as of October 31, 2023, in the Washington Disease Reporting System, 1,310 (0.07%) were missing data on age, 44,711 (2.4%) on sex, and 818,374 (43.6%) on race and ethnicity. Of 78,293 hospitalizations, 21 (0.03%) were missing data on age, 885 (1.1%) on sex, and 46,006 (58.8%) on race and ethnicity. Where information was missing, we assigned the median probability of developing PCC from the Household Pulse Survey. The probabilities provided by the Household Pulse Survey do not include people aged younger than 18 years. To estimate PCC incidence and prevalence among people younger than 18 years, we used the median probability of all age groups for experiencing PCC.

Because of the large uncertainties in multiple parameters, we incorporated parameter sampling into model iterations: we took the median value from the data, or fitting result, and sampled from values 25% smaller and larger than the median value (https://github.com/arranhamlet/long_covid_paper). We used these values in a Latin hypercube sampling (LHS), which allowed us to generate near-random samples of parameter values from a multidimensional distribution. We repeated this process 100 times to generate a broad measure of uncertainty in predictions, given variations in selected parameters. We then calculated the mean (95% CIs) of all LHS parameterization runs. 

### Statistical analysis

We conducted our analysis in R programming language and used Odin to code the mathematical model (28). All data and code required to run our analysis and produce figures are provided at https://github.com/arranhamlet/Long_covid_paper. This github repository contains walk-through examples of how to fit and run the model, produce plots, and analyze results.

## Results

### Model predictions approximate external benchmarks

**Figure 3:**
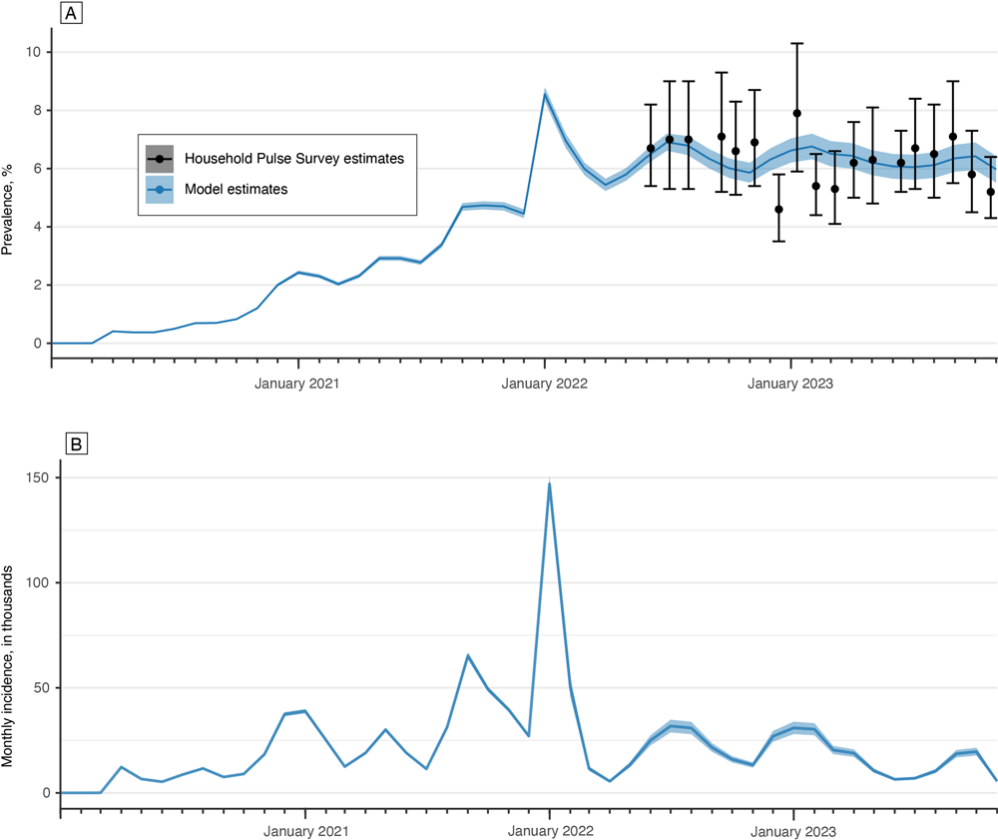
Prevalence and incidence of post–COVID-19 condition (PCC) among adults aged 18 years or older in Washington State, January 2020–October 2023. A) Model predictions (solid blue line) and Household Pulse Survey estimates (black circles) of PCC prevalence. Blue shading and error bars indicate 95% CIs. B) Estimated monthly incidence of PCC, in thousands. Shading indicates 95% CIs.

Model predictions of the prevalence of PCC among people aged 18 years or older in the general population in Washington State closely approximated the point prevalence estimates provided by the Household Pulse Survey to which they were fit (Figure 3A). Prevalence gradually increased during the study period, at different rates, dependent on COVID-19 incidence and pre-Omicron and post-Omicron periods, peaking in February 2023 at 8.6% (95% CI, 8.3%–8.8%). This prevalence fluctuated and remained elevated, with the latest estimate, in October 2023, indicating that 6.4% (95% CI, 5.9%–6.8%) of all adults in Washington State were experiencing PCC.

The incidence of PCC (Figure 3B) varied during the study period, peaking in January 2022 with a monthly incidence of more than 143,000 (95% CI, 139,000–147,000) new cases. The incidence decreased to a relatively low level, corresponding with a decreasing number of COVID-19 cases, with 5,500 (95% CI 4,900–6,000) new cases estimated in October 2023.

We estimated that 117,000 (95% CI, 67,000–182,000) of the adult population in Washington State had significant activity limitations because of PCC. This represents $1,762,956,000 (95% CI, $1,009,556,000–$2,742,376,000) in health care costs associated with PCC each year in Washington State.

### Substantial heterogeneity in PCC prevalence across age, sex, and race and ethnicity

**Figure 4:**
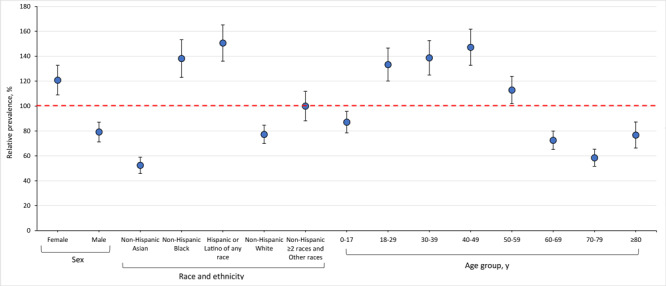
Estimated relative prevalence of post–COVID-19 condition, relative to the median, by sex, race and ethnicity, and age group among adults aged 18 years or older in Washington State in October 2023. The red dashed line indicates a prevalence ratio of 100% (no difference from the median prevalence). Error bars indicate 95% CIs.

Estimates of PCC prevalence were heterogeneous across subcategories in October 2023 (Figure 4). By age, the estimated relative prevalence of PCC, relative to the median, was highest among adults aged 18 to 29 years (133.3%; 95% CI, 120.1%–146.6%), 30 to 39 years (138.7%; 95% CI, 124.9%–152.6%), and 40 to 49 years (147.2%; 95% CI, 132.7%–161.7%) and lowest among people aged 60 to 69 years (72.5%; 95% CI, 65.1%–79.9%), 70 to 79 years (58.5%; 95% CI, 51.6%–65.3%), and 80 years or older (76.7%; 95% CI, 66.3%–87.2%). By sex, females were substantially more likely than males to have PCC in October 2023. The estimated relative prevalence of PCC, relative to the median, was 120.8% (95% CI, 109.0%–132.7%) among females and 79.2% (95% CI, 71.2%–87.1%) among males. By race and ethnicity, differences in the estimated relative prevalence of PCC were pronounced. The relative prevalence was lowest among people identifying as non-Hispanic Asian (52.4%; 95% CI, 45.8%–58.9%) and highest among people identifying as non-Hispanic Black (138.2%; 95% CI, 123.0%–153.3%) or Hispanic of any race (150.6%; 95% CI, 136.0%–165.2%).

**Table:** 

Characteristic	% (95% CI)
**Sex**	
Female	120.8 (109.0–132.7)
Male	79.2 (71.2–87.1)
**Race and ethnicity**	
Non-Hispanic Asian	52.4 (45.8–58.9)
Non-Hispanic Black	138.2 (123.0–153.3)
Hispanic or Latino of any race	150.6 (136.0–165.2)
Non-Hispanic White	77.2 (69.9–84.6)
Non-Hispanic ≥2 races and Other races	100.0 (88.2–111.8)
**Age group, y**	
0-17	87.1 (78.5–95.7)
18-29	133.3 (120.1–146.6)
30-39	138.7 (124.9–152.6)
40-49	147.2 (132.7–161.7)
50-59	112.9 (101.9–123.9)
60-69	72.5 (65.1–79.9)
70-79	58.5 (51.6–65.3)
≥80	76.7 (66.3–87.2)

### Estimated relative prevalence of PCC by county

**Figure 5:**
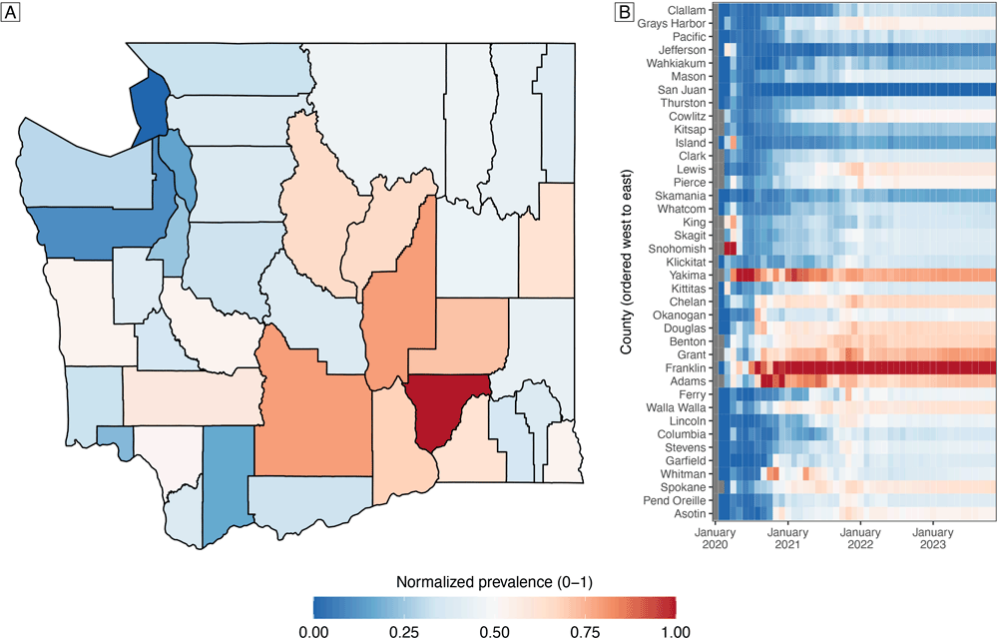
Estimated relative prevalence of post–COVID-19 condition (PCC), by county, Washington State. A) Map of the normalized prevalence of PCC in October 2023. B) Heatmap of counties normalized prevalence over time. The heatmap normalized prevalence values are specific to each time point to emphasize which counties at each time point were experiencing the highest PCC prevalence. Normalized prevalence is the prevalence recalculated on a 0-1 scale where 0 indicates the lowest prevalence and 1 the highest prevalence across all counties.

The normalized prevalence (on a scale of 0–1) allowed us to compare prevalence values across counties and periods (Figure 5). In October 2023, central Washington had the highest normalized prevalence in the state (Figure 5A). These trends remained relatively stable during the study period (Figure 5B).

## Discussion

We estimated that the prevalence of PCC among adults in Washington State was substantial as of October 2023, despite a reduction in COVID-19 cases during our study period. Although PCC prevalence varied over time and was lower in October 2023 than its peak in early 2022, it remained elevated. This persistent trend, which continued after the dominance of Omicron variants (13) and a decrease in levels of viral transmission, suggests that PCC will continue to have a substantial effect on the population in Washington State.

Heterogeneity in estimated PCC prevalence by subgroup was stark. Women, people aged 18 to 49 years, and people self-identifying as non-Hispanic Black or Hispanic appeared to have an elevated risk for PCC. These disparities do not necessarily indicate a biological difference and instead may represent generalized differences in socioeconomic determinants of health (29).

Individual demographic characteristics were not the only factors associated with PCC prevalence. Counties had substantially different patterns of PCC burden. Interventions and adherence to measures designed to reduce the spread of SARS-CoV-2 varied substantially among counties. Survivorship bias might also explain some of the differential burden of PCC. The lower relative prevalence of PCC among men and older age groups might have resulted from the association between these factors (age and sex) and death or care seeking (30,31). However, survivorship bias and trends in care seeking would not explain the trends we found among younger age groups or across racial and ethnic groups and is unlikely to have been the determining factors in the patterns we observed.

No single factor can explain the complicated mosaic of PCC burden in Washington State, but by incorporating each factor in our approach, we have a first glimpse into understanding it. Although we were unable to estimate the absolute prevalence and incidence of PCC in each subgroup, we did estimate relative burden, which will allow us to direct and prioritize further studies and interventions. This limitation (only being able to calculate relative rather than absolute prevalence) occurs because approximately 80% of symptomatic infections are not reported in the Washington State Department of Health surveillance system (22). By using estimates of unreported COVID-19 cases, we accounted for this surveillance gap. However, we did not have any information on these unreported symptomatic infections by age group, sex, race and ethnicity, or vaccination status. In our calculations of the relative prevalence estimates, if data for subgroups were missing at random and correlated with the true values, then the missing data will not have unduly biased our results. However, if the missing data were not random, then we may have propagated this bias through to our results. For example, to be counted as a confirmed COVID-19 case, a positive SARS-CoV-2 test is needed. However, differences in testing rates by race and ethnicity (32) may have led us to systematically underestimate or overestimate the true effect of PCC on certain groups. In addition, assigning the median value of a parameter (eg, age group, sex, race and ethnicity) in the absence of known subgroup information might have biased our results. For people aged younger than 18 years, the Household Pulse Survey does not collect information on PCC. Although some literature suggests that children are less likely than older age groups to develop PCC (33,34), in the absence of a quantified and comparable risk to other age groups in the Household Pulse Survey, we assigned the median value as the most parsimonious approach.

The use of mathematical modeling here allowed us to extrapolate beyond the available data and explicitly state our simplifications and assumptions in a logical framework. However, a model is only as good as the data on which it is based. Inaccuracies and biases in the data and the assumptions made in the model formulation carry forward into results. Although mathematical modeling is an important tool in public health decision making, it serves to enhance, not replace, the collection and interpretation of high-quality data.

To compensate for the shortcomings of the data and the general uncertainty about parameters, we used the LHS approach. This approach — taking a wide distribution of parameter values around a central mean — introduced robustness to model estimates. Because our results already accounted for a range of parameter values and variation, changes to individual parameters would have a minimal effect on the magnitude or direction of our results.

### Limitations

A limitation of our study was the inability to use independent data to assess the model fit data. No estimates of PCC prevalence in Washington State are available other than estimates provided by the Household Pulse Survey. However, even when estimates of PCC prevalence exist, there are problems with comparison. Research on PCC is at an early stage, and a comprehensive understanding is needed of what PCC encompasses and its underlying mechanisms (5). Early research has resulted in multiple definitions and approaches (21). Definitions differ on PCC symptoms, how symptoms change over time, and how long they are experienced, and differences will likely persist as we learn more. Although alternative indicators of the effects of PCC might emerge (eg, effect on the workforce and disability claims), we have been unable to find representative data sets to compare model outputs. However, should alternative data sources become available, the framework described here allows for their easy implementation.

Although our model accounted for numerous factors that have been shown to influence the probability of developing PCC, because of the inherent limitations of working in a new and expanding field, we cannot account for all of them. Evidence exists of multiple aspects that might change a person’s risk for PCC, including reinfection (11) and preexisting conditions (5) that we have not been able to include for lack of data. Similar problems exist in the designation of our subgroups, particularly with data on race and ethnicity, which we acknowledge is a social construct that often represents a coarse aggregation of a diverse population. Additionally, although we included a pre-Omicron and post-Omicron change in the proportion of symptomatic infections and the probability of developing PCC, this inclusion did not fully capture the diversity of variants experienced. The omission of these factors might affect the overall and subgroup model estimates. However, in the absence of either individual-level data or parameter estimates from literature, we were unable to account for these factors.

### Conclusion

The estimation of PCC prevalence is an important first step in understanding the effects of PCC as a public health problem. However, work still needs to be done to translate this prevalence into the effects felt by the population. Although long-term disability appears to occur in only a minority of people with PCC, the number of PCC cases means that PCC could impose a considerable burden on the health care system. The effects of PCC have both social and financial costs. In addition to the direct health care costs of managing PCC, indirect costs are also incurred. An estimated 56% of persons with PCC who previously worked had either reduced their hours or were unemployed (35). Our study, by accounting for heterogeneous subgroups and geographies, aimed to highlight disparities in effects across Washingtons State to guide awareness and relief efforts.

We are still in the early stages of understanding the long-term implications of SARS-CoV-2 infection and PCC. Although promising therapeutic treatments are on the horizon (5), much must be done to understand the underlying mechanisms of PCC, identify groups at risk of PCC, and develop effective interventions to alleviate the burden of PCC.
